# The prevalence and anatomy of recurrent artery of Heubner: a meta analysis with neurosurgical considerations

**DOI:** 10.1007/s00701-024-06327-0

**Published:** 2024-10-29

**Authors:** Aleksander Osiowski, Kacper Stolarz, Maksymilian Osiowski, Tomasz Klepinowski, Dominik Taterra

**Affiliations:** 1 Faculty of Medicine, Jagiellonian University Medical College, Sw. Anny 12, 31-008 Krakow, Poland; 2 Department of Neurosurgery, Pomeranian Medical University Hospital No, 1, Unii Lubelskiej 1, 71-252 Szczecin, Poland; 3 Department of Orthopedics and Rehabilitation, Jagiellonian University Medical College, Balzera 15, 34-500 Zakopane, Poland; 4 Ortho and Spine Research Group, Zakopane, Poland

**Keywords:** Recurrent artery of Heubner, Anatomy, Distal medial striate artery, Anterior cerebral artery, Circle of Willis

## Abstract

**Background:**

The recurrent artery of Heubner (RAH) is typically the largest medial lenticulostriate branch of the anterior cerebral artery (ACA). Neurosurgical procedures such as aneurysm treatment on the anterior part of the circle of Willis can result in damage of the RAH leading to neurological deficits. The aim of this study was to identify the gaps and provide comprehensive data on the prevalence and anatomical characteristics of the RAH with neurosurgical considerations.

**Methods:**

The major electronic databases were thoroughly searched to identify the eligible studies. The information concerning study type, geographical origin, prevalence of the RAH, course and origin of the RAH, symmetry of origin and number of RAHs in each hemisphere, and morphometric data were extracted. The PRISMA guidelines were rigorously followed throughout the study. The AQUA tool was used to evaluate the reliability of included studies.

**Results:**

A total of 34 studies (*n* = 3645 hemispheres) were included in the meta-analysis. The analysis revealed that the RAH was present in 97.5% (95%CI: 95.5–98.6) of the hemispheres, originating most frequently from the A2 segment (42.2%, 95%CI: 35.0–49.7) or the ACoA-ACA junction (41.6%, 95%CI: 34.0–49.6), and coursing anteriorly (47.6%, 95%CI: 38.7–56.6) or superiorly (43.9%, 95%CI: 34.4–53.8) in relation to ACA. Almost a quarter of patients had more than one RAH, which was on average 22.82 mm (SD: 1.35, 95%CI: 20.16–25.47; I^2^ = 99.1%, *p* < 0.01) long and reached 0.76 mm (SD: 0.05, 95%CI: 0.66–0.85; I^2^ = 99.4%, *p* < 0.01) in diameter.

**Conclusions:**

As the RAH is present in the majority of the population, it is important to be aware of the wide variations in its anatomy. This will help to prevent postoperative neurological deficits by avoiding undesirable complications during surgeries that are performed in close proximity to the anterior segment of the circle of Willis.

**Supplementary Information:**

The online version contains supplementary material available at 10.1007/s00701-024-06327-0.

## Introduction

The recurrent artery of Heubner (RAH), also named as distal medial striate artery, was first described by the German pediatrician Johann Otto Leonhard Heubner in 1872 [26] as an artery arising from theanterior cerebral artery (ACA) and supplying blood to the anterior part of the caudate nucleus, the anterior third of the putamen, the anterior limb of the internal capsule and the globus pallidus [13]. The artery also supplies the olfactory region, the anterior hypothalamus, the nucleus accumbens, the diagonal band of Broca, parts of the uncinate fasciculus and the basal nucleus of Meynert [25, 40, 41, 53, 70]. The RAH arises from proximal A2 segment of the ACA, the junction of ACA and the anterior communicating artery (ACoA) or less often the A1 segment of ACA and is usually the largest of the medial lenticulo-striate arteries branching from ACA [13]. The RAH takes a lateral course towards the anterior perforated substance (APS), extending over the level of the internal carotid artery (ICA) bifurcation and straight gyrus [63].

Surgical procedures such as aneurysm treatment on the anterior part of circle of Willis can result in damage or occlusion of the RAH [66], which can lead to hemiparesis with facial and brachial predominance or aphasia if the artery is on the dominant side. Moreover, iatrogenic lesion of RAH may occasionally result in significant neuropsychological deficits, such as personality changes, memory loss and impairment of cognitive functions [14, 20, 34, 40, 53]. The development of these symptoms particularly finds explanation in the supply territory of RAH and other basal perforating arteries originating from ACoA complex, which may involve crucial parts of the limbic system (e.g. substantia innominata) [14, 20].

The majority of our current knowledge regarding the anatomy and morphology of RAH can be attributed to numerous anatomical studies [13, 19, 20, 34–36, 39, 70]. However, the collected data remain inconclusive and inconsistent, with significant discrepancies in relation to prevalence, course, origin, number, symmetry and morphology of RAH. Additionally, the majority of anatomical studies are based on small groups of specimens, while reports based on larger numbers of specimens are scarce. Therefore, understanding the intricate anatomical variations of the RAH is crucial for neurosurgeons to prevent undesirable iatrogenic complications [14, 20, 34, 40, 53] and thus, a comprehensive unification of the available evidence is required. As such, the aim of this study was to analyze the main characteristics and examine the clinical anatomy of RAH by thorough investigation of available data on its prevalence, origin, course, and morphology using a systematic approach.

## Methods

### Search strategy

The major electronic databases (Pubmed, EMBASE, Web of Science, and ScienceDirect) were searched for studies on the RAH. The search terms included: recurrent artery of Heubner OR Heubner OR medial striate artery OR long central artery. No date and language limits were applied. To identify additional eligible studies, a reference search was performed. The Preferred Reporting Items for Systematic Reviews and Meta-analyses [PRISMA] [49] guidelines were followed in this study.

### Eligibility assessment

Two independent authors assessed the eligibility of the study for the inclusion in the meta-analysis. All cadaveric, intraoperative and imaging studies were included. The exclusion criteria contained: articles published as case series, case reports, review articles, letters to editor, conference abstracts, animal studies, and articles containing irrelevant or incomplete data. We also included studies written in languages other than English. Articles that were in languages other than English were translated by medical professionals fluent in English and the article’s language. Any inconsistencies between authors during eligibility assessment were solved by consensus among all reviewers.

### Data extraction

Data extraction was conducted by 2 independent authors. Data regarding the type of included studies (cadaveric, imaging and intraoperative), region of origin, overall prevalence of RAH in population and characteristics: course of RAH (in relation with A1 segment of the ACA)—anterior, superior, posterior and inferior course, origin (A1 segment, A2 segment or AcoA-ACA junction), symmetry of origin and number of RAHs in each hemisphere, length and diameter. Studies that included data of mean length and diameter of RAH with standard deviation (SD) were included in this subgroup analysis. If SD was missing, the proper estimation was applied (95%CI/4). The authors of original articles were contacted if any necessary or additional information was needed.

### Quality and risk of bias assessment

The AQUA tool [23] was used by the reviewers to evaluate the quality and reliability of the included studies. In brief, the tool was devised in order to probe for potential bias in anatomical studies. Five domains were evaluated in the analysis: (1) objective(s) and subject, (2) study design, (3) methodology characterization, (4) descriptive anatomy, and (5) reporting of results. Each domain was determined as either "low," "high," or”unclear” risk of bias [23]. A decision was made that a “no” answer in whichever signaling question within each of the categories made the domain to be of “high” risk of bias, whereas all answers “yes” suggested that it presented a “low” risk of bias [23]. The "unclear” option was chosen when the study data did not permit clear assessment.

### Statistical analysis

Statistical analysis was performed using MetaXL version 5.8 by EpiGear Pty Ltd. (Wilston, Queensland, Australia). A random-effects model was used for all statistical analyses. To assess the heterogeneity between the included studies, the Chi^2^ test and the I^2^ statistic were used. For the Chi2 test, the p-value of Cochran's Q was < 0.10 and indicated significant heterogeneity of included studies. The results of the I^2^ statistic were considered: 0–40% as could not be important, 30–60% as could indicate moderate heterogeneity, 50–90% as could indicate substantial heterogeneity, and 75–100% as could represent considerable heterogeneity” [27].

## Results

### Study identification and characteristics of included studies

**Fig. 1:**
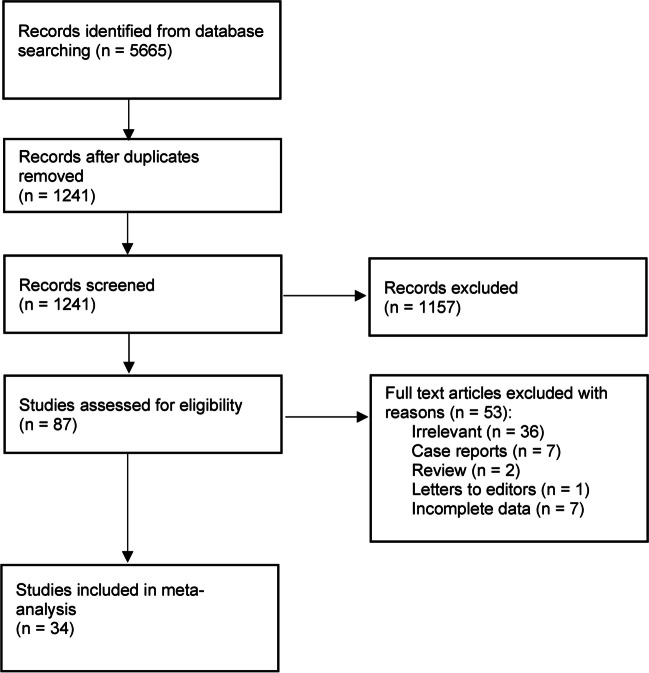
Study flow diagram

The study selection process is presented in Fig. 1. The initial search through electronic databases resulted in 5665 entries. The analysis of 87 full-texts resulted in exclusion of 53 studies. Finally, a total of 34 [2–6, 9–13, 16, 19, 20, 29, 31, 34, 36, 38, 39, 43, 44, 46, 51, 53, 55, 57, 59–64, 69, 70] studies were included in this meta-analysis.

The characteristics of included studies are presented in Table 1. A total of 34 studies published between 1967 and 2024 were included. The studies had diverse geographical origin with 11 studies originating from North America, 3 from South America, 9 from Asia and 11 from Europe.

**Table 1:** Characteristics of included studies

Study	Country	Type of study	Number of subjects (hemispheres)	% Prevalence of RAH^a^ (# subjects with RAH)
Agosti 2022 [2]	USA	cadaveric	16	100 (16)
Agrawal 2019 [3]	India	intraoperative	62	71 (44)
Ahmed 1967 [4]	England	cadaveric	24	91.7 (22)
Avci 2003 [5]	Turkey	cadaveric	62	98.4 (61)
Aydin 1994 [6]	Turkey	intraoperative	96	100 (96)
Boongird 2009 [9]	Thailand	cadaveric	100	100 (100)
Corredor 2020 [16]	Colombia	cadaveric	142	96.5 (137)
DAvella 2015 [10]	Austria	cadaveric + radiologic	20	100 (20)
Dimitriu 2013 [11]	Romania	cadaveric + radiologic (DSA)	224	94.6 (212)
Dunker 1976 [12]	USA	cadaveric	40	70 (28)
ElFalougy 2013 [13]	Slovakia	cadaveric	366	94.8 (347)
Gasca Gonzalez 2017 [46]	Mexico	cadaveric	30	93.3 (28)
Gomes 1984 [19]	USA	cadaveric	60	96.7 (58)
Gorczyca 1987 [20]	Canada	cadaveric	100	100 (100)
Kedia 2013 [29]	India	cadaveric	30	100 (30)
Lemos 1976 [31]	Brasil	cadaveric	166	98.8 (164)
Loukas 2006 [34]	Dutch Antilles	cadaveric	69	94.2 (65)
Maga 2013 [36]	Poland	cadaveric	140	98.6 (138)
Marinkovic 1986 [38]	Serbia	cadaveric	66	100 (66)
Matsuda 2018 [39]	Japan	cadaveric	714	98.7 (705)
Musso 2002 [43]	Brasil	cadaveric	100	100 (100)
Najera 2019 [44]	USA	cadaveric	50	100 (50)
Papazova 2018 [51]	Macedonia	cadaveric	266	94 (250)
Perlmutter 1976 [53]	USA	cadaveric	100	99 (99)
Rosner 1984 [55]	USA	cadaveric	50	100 (50)
Tao 2006 [57]	China	cadaveric	90	100 (90)
Tulleken 1978 [59]	Netherlands	cadaveric	150	100 (150)
Ugur 2006 [60]	Turkey	cadaveric	100	100 (100)
Uzun 2009 [61]	Turkey	cadaveric	108	100 (108)
Valli 2021 [62]	USA	cadaveric	10	100 (10)
Vasovic 2009 [63]	Serbia	cadaveric	188	97.3 (183)
Vasquez Loayza 2016 [64]	USA	cadaveric	80	58.8 (47)
Zhu 2021 [69]	China	cadaveric	72	95.8 (69)
Zunonkipre 2011 [70]	France	cadaveric	40	100 (40)

*RAH* recurrent artery of Heubner

### Quality and risk of *bias* assessment

The majority of studies included in this meta-analysis, evaluated by the AQUA tool, revealed domain three (methodology characterization) to be at “High” risk of bias, owing it most to no information regarding experience of the researchers. A considerable amount of studies revealed domain one (objective(s) and subject characteristics) to be at “High risk of bias, owing it mainly to missing demographic data of the research group. All studies had a “Low” risk of bias found in domain two (study design). Almost all studies had a “Low” risk of bias in domain four (descriptive anatomy) and domain five (reporting of results) (Supplement 1).

### Prevalence of the recurrent artery of Heubner

**Fig. 2:**
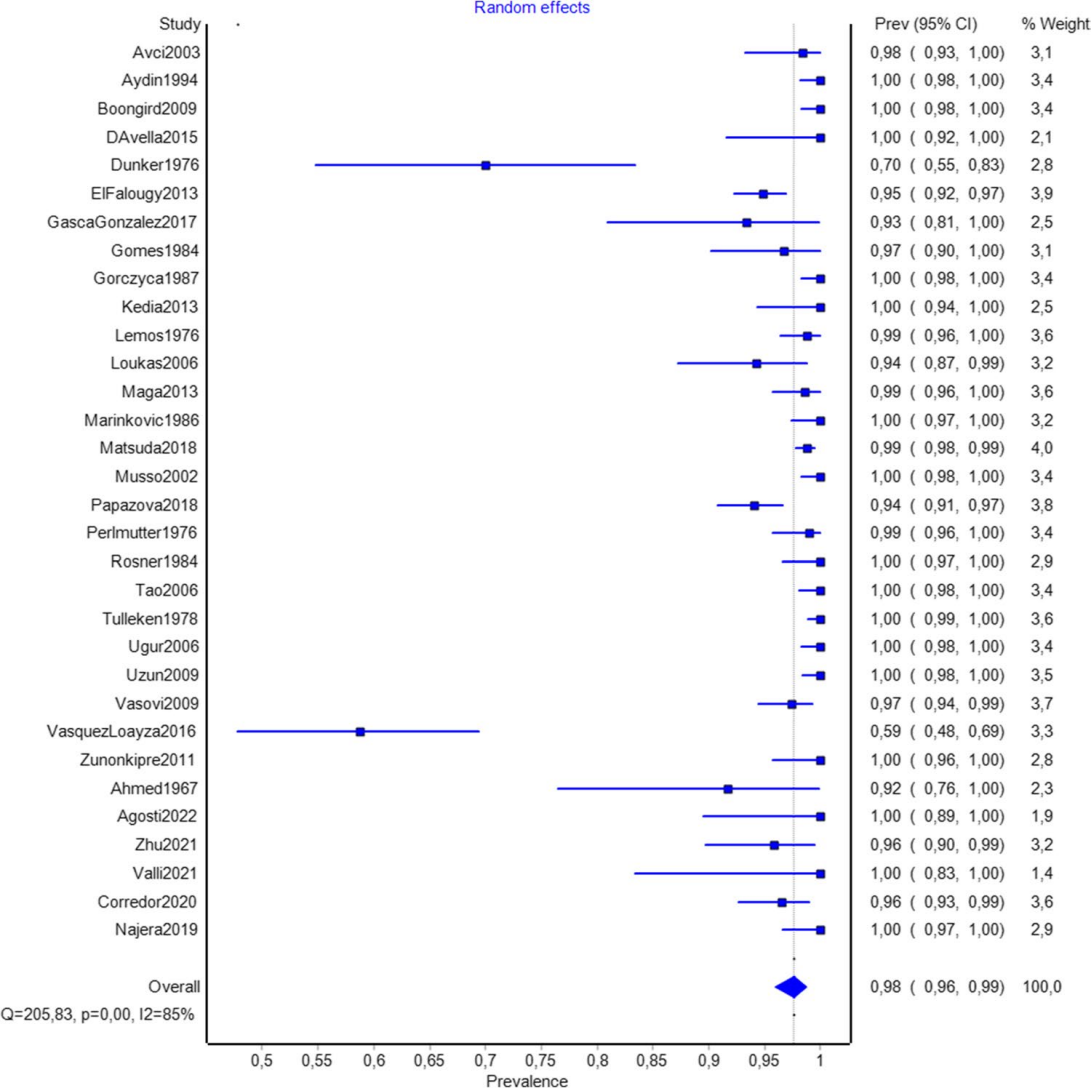
A forest plot depicting global pooled prevalence of the recurrent artery of Heubner

A total of 32 studies (*n* = 3,645 hemispheres) were included in the analysis on the prevalence of RAH (Fig. 2). Pooled prevalence estimate (PPE) of RAH was 97.5% (95%CI: 95.5–98.6). The subgroup analysis on the prevalence of RAH per brain (30 studies, *n* = 1,780 brains) showed a PPE of 97.4% (95%CI: 95.1–98.6) (Table 2).

**Table 2:** Overall prevalence of recurrent artery of Heubner

Subgroup		Number of studies (number of subjects)	Pooled prevalence of RAH: % (95% CI)	I^2^: %	Cochran’s Q, p-value
Overall per hemispheres		32 (3645)	97.5 (95.5–98.6)	85.97	< 0.001
Overall per brains		30 (1780)	97.4 (95.1–98.6)	69.99	< 0.001
Type of study	Cadaveric	31 (3549)	97.4 (95.4–98.6)	86.21	< 0.001
Region of origin	Asia	9 (1372)	98.9 (97.1–99.6)	0.0	0.602
	Europe	9 (1260)	97.1 (93.6–98.7)	38.77	0.110
	North America	11 (605)	93.6 (87.1–97.0)	86.19	< 0.001
	South America	3 (408)	98.3 (93.3–99.6)	32.97	0.225

*RAH* recurrent artery of Heubner

The analysis of 31 cadaveric studies (*n* = 3,549 hemispheres) revealed a prevalence rate of RAH of 97.4% per hemisphere (95%CI: 95.4–98.6). The studies that originated in North America showed the lowest pooled prevalence rate of RAH per hemispheres (93.6%, 95%CI: 87.1–97.0), followed by studies from Europe (97.1%, 95%CI: 93.6–98.7), South America (98.3%, 95%CI: 93.3–99.6) and Asia with the highest PPE (98.9%, 95%CI: 97.1–99.6), although the differences in PPE between the continents were not statistically significant (Table 2).

### Course of the recurrent artery of Heubner

**Fig. 3:**
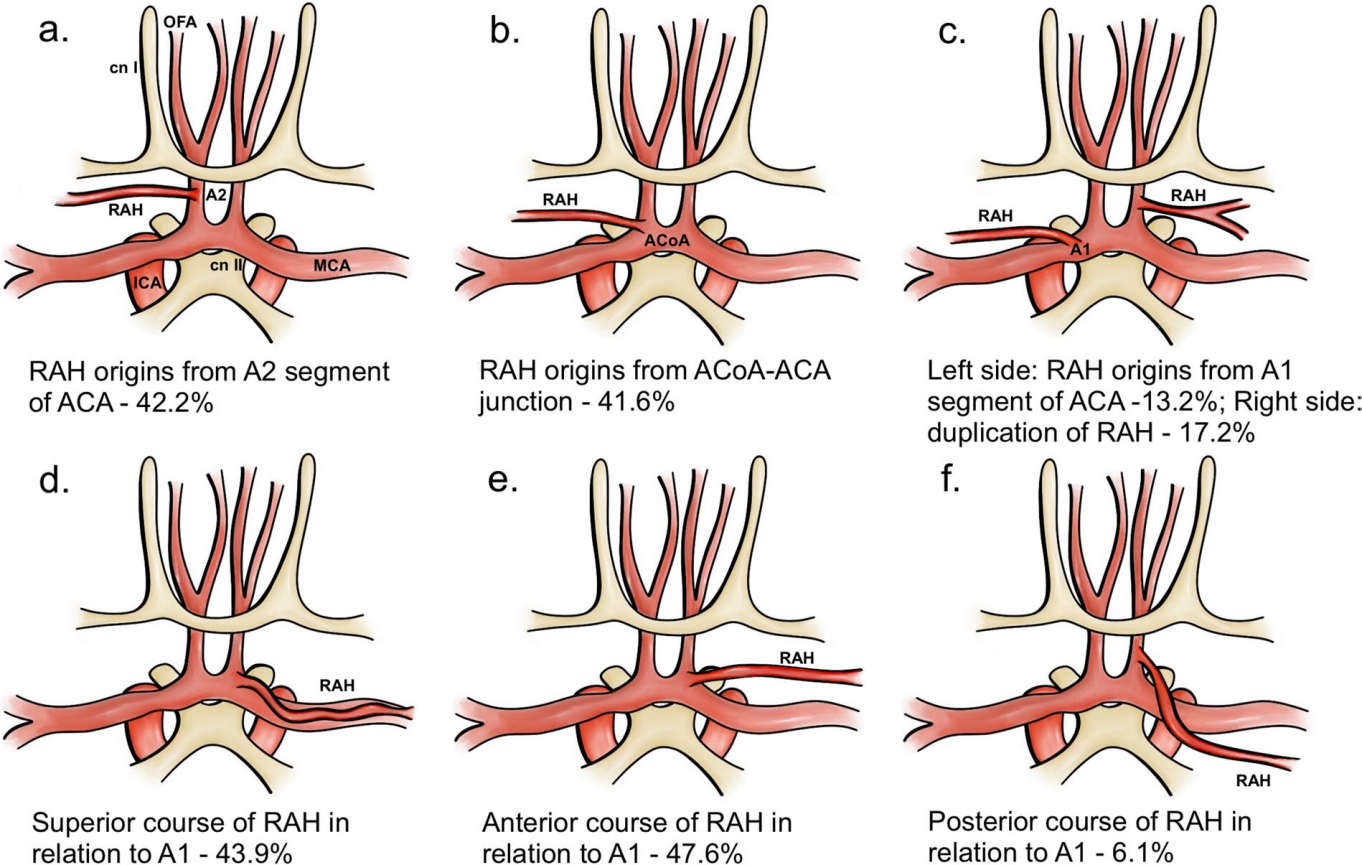
Illustrative presentation of the most common types of the recurrent artery of Heubner. Panels **a**, **c**, **b** show origin sites of RAH, panels **d**, **e**, **f** show most common course patterns of RAH. RAH = recurrent artery of Heubner, OFA = orbitofrontal artery, cn I = first cranial nerve (olfactory nerve), cn II = second cranial nerve (optic nerve), MCA = middle cerebral artery, ICA = internal carotid artery, ACA = anterior cerebral artery, ACoA = anterior communicating artery

A total of 14 studies (*n* = 2,284 RAHs) were included in the analysis on the course of RAH (Table 3). Both superior course and anterior course of RAH, in relation to the A1 segment of ACA, were the most frequent course patterns observed. There were no significant differences between PPE of two course patterns, with RAH coursing superiorly in 43.9% (95%CI: 34.4–53.8) of cases and anteriorly in 47.6% (95%CI: 38.7–56.6) of cases. The artery ran posteriorly to ACA significantly less often with PPE of 6.1% (95%CI: 3.9–9.3) and inferiorly in 0.8% of cases (95%CI: 0.4–1.7) (Table 3 and Fig. 3).

**Table 3:** Course of recurrent artery of Heubner (RAH) in relation to anterior cerebral artery (ACA)

Number of studies (number of RAH)	Superiorly to ACA % (95% CI)	Anteriorly to ACA % (95% CI)	Posteriorly to ACA % (95% CI)	Inferiorly to ACA % (95% CI)	I^2^: %	Cochran’s Q, p-value
14 (2284)	43.9 (34.4–53.8)	47.6 (38.7–56.6)	6.1 (3.9–9.3)	0.8 (0.4–1.7)	96.11	< 0.001

### Origin of recurrent artery of Heubner

A total of 28 studies (*n* = 3,839 RAHs) analyzed the origin of RAH (Table 4). The artery originated most frequently from the A2 segment with PPE of 42.2% (95%CI: 35.0–49.7), followed by ACoA-ACA junction with PPE of 41.6% (95%CI: 34.0–49.6). The origin from the A1 segment was the least frequent with PPE of 13.2% (95%CI: 9.9–17.4) (Table 4) (Fig. 3).

**Table 4:** Origin of RAH

Number of studies (number of RAH)	A1 segment % (95% CI)	A2 segment % (95% CI)	ACoA-ACA junction % (95% CI)	I^2^: %	Cochran’s Q, p-value
28 (3839)	13.2 (9.9–17.4)	42.2 (35.0–49.7)	41.6 (34.0–49.6)	96.7	< 0.001

*RAH* recurrent artery of Heubner, *ACoA *anterior communicating artery, *ACA *anterior cerebral artery

The analysis of studies reporting the data on the symmetry of origin of RAH in the brains showed that the RAH had asymmetrical origin in both hemispheres in 46.9% (95%CI: 34.8–59.4; I^2^ = 83.0%, *p* = 0.00) of cases, while in 53.1% (95%CI: 40,6–65,2; = 83.0%, *p* = 0.00) of cases, the RAH origin in both hemispheres was symmetrical.

### Number of recurrent arteries of Heubner

A total of 26 studies (*n* = 3,503 hemispheres) analyzed the number of RAHs per hemisphere. In 76.0% (95%CI: 65.9–83.9) of the hemispheres, only one RAH was observed. Duplication of RAH was seen in 17.2% (95%CI: 11.9–24.4) (Fig. 3) of hemispheres, triplication in 2.5% (95%CI: 1.4–4.7) of hemispheres and quadruplication in 1.0% (95%CI: 0.7–1.6) of hemispheres. The artery was absent in 2.9% (95%CI: 2.1–3.9) of hemispheres (Table 5).

**Table 5:** Number of recurrent arteries of Heubner (RAHs) per hemisphere

Number of studies (number of hemispheres)	0 arteries % (95% CI)	1 artery % (95% CI)	2 artery % (95% CI)	3 arteries % (95% CI)	4 arteries % (95% CI)	I^2^: %	Cochran’s Q, p-value
26 (3503)	2.9 (2.1–3.9)	76.0 (65.9–83.9)	17.2 (11.9–24.4)	2.5 (1.4–4.7)	1.0 (0.7–1.6)	97.2	< 0.001

The analysis of laterality of RAH (6 studies, *n* = 350 brains with bilateral RAH) revealed that the single bilateral RAH was present in 75.6% (95%CI: 56.9–87.9; I^2^ = 84.6%, *p* = 0.00) of the brains making it the most common laterality pattern. In 13.4% (95%CI: 5.9–27.5; I^2^ = 77.9%, *p* = 0.00) of the brains RAH was doubled unilateraly, while bilateral duplication of RAH was reported in 9,9% (95%CI: 4.2–21.6; I^2^ = 74.8%, *p* = 0.00) of the examined brains.

### Morphometric analysis of recurrent arteries of Heubner

A total of 11 studies (*n* = 1,197 RAHs) analyzed the length of RAH. The calculated mean length of the artery was 22.82 mm (SD: 1.35, 95%CI: 20.16–25.47; I^2^ = 99.1%, *p* = 0.00). A total of 15 studies (*n* = 2,557 RAHs) analyzed the diameter of RAH. The calculated mean diameter of the artery was 0.76 mm (SD: 0.05, 95%CI: 0.66–0.85; I^2^ = 99.4%, *p* = 0.00).

## Discussion

The aim of the study was to provide a detailed description of the anatomy and morphology of RAH through a systematic analysis of existing literature. In this meta-analysis, we have found that the RAH is present in nearly all human specimens studied, averaging at 97.4% pooled prevalence, with varying origins, courses through the cranium, and symmetry. Our study showed that the RAH runs as a single artery in around 76.0% of the patients and can be duplicated in 17.2% of the patients. Triplication, quadruplication, and absence of RAH may be noted in smaller percentages of the cases. It is present mostly bilateral and courses anteriorly and superiorly to ACA, with a small portion also coursing inferiorly or posteriorly in relation to the ACA. It is also varied in its origin. According to Perlmutter and Rhoton [53], RAH arises in close proximity to ACoA, as ninety-five percent of RAHs are detectable within 4 mm of ACoA, either proximal or distal. Our findings showed that the A2 segment and the ACoA-ACA junction are the most common places of origin, and the A1 segment is the least common origin. As indicated by results of our study, the anatomy of the RAH is diverse in nearly every aspect of its anatomy.

There are four groups that comprise the perforating branches of the circle of Willis [21]. The anteromedial group descends from A1 and ACoA and subsequently supply the structures in the area of anterior part of hypothalamus and optic chiasm [21]. Arteries that arise from M1 and A1 represent the anterolateral group of the perforating branches [21]. Lenticulo-striate arteries (LSA), belonging to the anterolateral group, enter the hemisphere through the APS [21]. The recurrent artery of Heubner, which is generally the largest of the medial LSA branches of the ACA [34], is a representative of the anterior perforating artery group. Branches from internal carotid artery and anterior choroidal artery enter APS in its posterior half portion, branches from M1 and M2 segments enter the middle and posterior parts of the lateral APS, branches from A1 enter the medial half of the APS and lastly RAH, which may enter as a single vessel or as divided branches [9], enters the anterior two-thirds of the mediolateral part of APS [48, 55]. They all share a common feature of penetrating the APS [55] and supplying the same brain region of basal ganglia, strongly supporting the theory of a common embryological origin [48]. The middle cerebral artery (MCA) shares the same embryologic origin as RAH, as both of them are derived from the embryological lateral striate artery and may supply equal brain areas [7, 18, 37]. According to Abbie’s phylogenetic concept [1], RAH is the survivor of the anastomoses over and around the paleo-olfactorium. Moreover, besides ACA being the main blood supplier to RAH, it may still retain its prenatal MCA connections. The existence of anastomosing perforating branches between MCA and RAH, along with the wide variations in RAH’s blood supply [7], play in favor of Abbie’s statement [1, 19, 20, 35, 56, 70].

In 1960, Ostrowski et al. [47] published the first paper that provided a thorough description of RAH parenchymal supply. More than six decades later, it continues to be recognized that RAH constantly supplies the anterior part of the caudate nucleus, the anterior third of the putamen, the anterior part of the outer segment of the globus pallidus, the anteroinferior portion of the anterior limb of the internal capsule, the uncinate fasciculus, the olfactory region, the anterior hypothalamus, the diagonal band of Broca, the basal nucleus of Meynert, and the nucleus accumbens. Moreover, the RAH perfuses paleocortex and diencephalic regions [9, 12, 14, 19, 20, 25, 28, 34, 40, 41, 53, 63, 70].

Considering the branching pattern of RAH, the ones being constantly observed in the literature are the following branches: olfactory, frontal, tiny hypothalamic, lateral fissure, APS, and orbito-frontal branches (different from medial orbitofrontal artery) [5, 19, 20, 53, 63, 70]. Perlmutter and Rhoton [53] reported that none of RAH branches supplied the optic chiasm or optic tract. Additionally, a few uncommon occurrences were seen [63]: anastomotic loops between two RAH, fenestration of a single RAH, and pseudofenestration of the A1 segment by a piercing ipsilateral RAH.

In order to mitigate the incidence of iatrogenic complications, it is crucial for neurosurgeons operating in the proximity of the anterior half of the circle of Willis to recognize and identify anatomical variations of the RAH, which typically originates few millimeters proximal or distal to ACoA area as our study showed. This region of the circle of Willis is where aneurysm formation becomes particularly common, representing about 30% of all cerebral aneurysms [50]. Iatrogenic damage to RAH may cause mediobasal striatum infarction [70], which leads to brachiofacial hemiparesis and aphasia [19, 34, 50], with motivational and emotional symptoms [14, 20, 34, 40, 53]. Injuries to RAH can additionally include symptoms like tongue and palate dysfunction, which can be observed during a careful swallowing evaluation [65]. Additionally, expressive aphasia may arise from involvement of the dominant hemisphere [8, 22, 30, 33, 52, 65]. The wide variation in the clinical presentation of infarctions in this region is likely due to the significant overlap of the vascular territories of the deep perforators originating from the ACA and MCA [17, 41]. Results of the study reported by Feekes et al. [15] suggest a very consistent branching pattern and spatial organization for the basal ganglia microvascular domains. The basal ganglia vasculature contain few anastomoses between the large parental vessels or their major branches [15, 32]. Moreover, the main penetrators, LSA and RAH, lack web-like anastomoses [15]. Thus, even in rare cases where there are minor deviations from the standard course of the penetrating vessels (such as abnormalities in the morphology and course of the RAH), the microvascular bed remains spatially unchanged, and the vascular territories of distinct arteries do not overlap significantly [15]. We conclude that this could account for the similar clinical picture of the RAH infarction regardless of the anatomical variation.

We have also demonstrated that the RAH arises predominantly from the A2 segment (42.2% of the analyzed arteries), taking a frontobasal course towards the APS, superiorly and anteriorly in relation to the proximal A2 segment of ACA in over 95% of cases. Many authors suggest [36, 70] that in order to prevent postoperative neurological deficits, it is advisable that the surgeon should routinely identify and avoid unnecessary manipulations [20] of RAH during surgeries involving ACoA aneurysm clipping, especially considering that RAH may be the first vessel encountered during a subfrontal dissection along the superior aspect of the optic nerve [42, 53]. During aneurysm surgeries revolving around the anterior half of the circle of Willis, distinguishing between the RAH and the orbitofrontal artery might serve quite a challenge [42]. One way of managing this difficulty would be observing their courses, as RAH usually follows the A1 segment, while the orbitofrontal artery would usually course across the olfactory tract as well as perpendicularly over the gyrus rectus [53]. Also, RAH could be mistaken with the accessory middle cerebral artery which arises from A1 or A2 ACA and initially has similar retrograde course but gives cortical branches [54].

During dissection or clipping the aneurysmal neck occurring in A1 segment of ACA and in ACoA with a posterior projecting dome, Gomes et al. [19] and Nathal et al. [45] suggested that a greater care needs to be applied for preservation of perforating vessels (especially RAH) in comparison to aneurysms with anterior projection, due to more difficult exposure of the area during the surgery. Moreover, Loukas et al. [34] found that RAH is frequently adhering to its originating artery, even up to 10 mm of the course, and emphasized that in such cases, RAH would be more vulnerable to damage during surgical treatment of the aneurysm of the parent vessel. Gomes et al. [19] advised that in cases of RAH involvement in the dome of the aneurysm, section and reimplantation of the artery into A1 segment should be considered. Similarly, when an atherosclerotic plaque obstructs the RAH's origin, the same protocol ought to be followed [19].

The uncommon prevalence of the posterior course of RAH (6.1%) may result in missing the artery during the surgery. Therefore, undetected RAH may be prone to injury during aneurysm clipping [70]. If the common path of the artery is not seen, a gentle dissection on the parent vessel's posterior surface is recommended [36] utilizing an endoscope for inspection. In order to expose this aneurysm, the posterior portion of the gyrus rectus may be resected in challenging cases. This maneuver, however, increases the risk of injuring the posteriorly coursing RAH [9, 20, 28]. Furthermore, many authors suggest that especially in both anterior and superior courses of RAH, the adventitia-arachnoid adherence with strands should be thoroughly sharply dissected [34, 36, 70].

According to Maga et al. [36], the number of RAHs carries a clinical significance itself. Result of this study shows that despite the fact that in three-quarters of cases RAH runs as a single vessel, double RAH can be present in every fifth patient. Moreover, even though triple and quadruple RAH variants may be found in over 3% of cases, their considerable presence neither can be neglected, nor ruled out. Regarding our findings, it is highly reasonable to suggest that once RAH is identified during the surgery, one should look for the presence of a second vessel in order to prevent any undesirable iatrogenic damage to an unidentified additional variant of RAH [36]. Interestingly, the presence of a double variant of RAH may be associated with other cerebrovascular variants and malformations [38]. Moreover, according to findings in Maga et al. [36] study and those reported by Gorczyca and Mohr [20], there is an inverse correlation between the number of arteries from the medial group of the LSA and the number of RAHs.

Our results showed that in rare cases, RAH may run posteriorly in relation to A1 segment. Therefore, missing posteriorly running RAH may result in its damage during the clipping of A1. By the same token, as Bonasia et al. [8] and Matsuda et al. [39] concluded, in sellar and parasellar surgery, and particularly in the pterional, subfrontal, or interhemispheric approaches to any aneurysm in the ACoA and ACA complex, RAH should be identified and careful microdissection of RAH, ACA, and ACoA is encouraged [67, 68].

Unification of data was especially important as significant differences can be seen in a number of previous series. For instance, the mean number of RAHs per hemisphere may vary from 0.7 to 2.0 between some cohorts [20]. Despite this, our study and all prior reports concur that it is extremely uncommon for there to be no RAH in a hemisphere [39]. Discrepancies can be also found in regard to the controversial major site of origin of RAH, as some studies reported pure dominance in the A1 segment, others in ACoA junction, while others in A2 segment [20, 39]. Additionally, the same follows with the double, triple and quadruple RAH variants [20]. Our study is the first to provide quantitative consensus in all of these major disputable topics. The results of this study also indicate that the true absence of RAH is fairly uncommon (2.9%), which justifies why it is crucial to attempt to locate this vessel prior to the clipping of ACoA aneurysm [70]. Feekes and Cassel [14] demonstrated that RAH could be efficiently identified preoperatively with thin slice CT angiography and according to our results, it should appear as a single artery in 76% of cases.

The limitation of our study was a considerable degree of heterogeneity among the included studies. Despite conducting subgroup analyses to probe the cause of the heterogeneity, it remained constant during the investigation. However, high heterogeneity is expected in this type of meta-analysis due to intrinsic heterogeneity of anatomical studies [24]. Another drawback is that the study protocol was not registered prior to this systematic review and meta-analysis. Although it is recommended, the global survey showed it was not a common practice [58]. Also, most studies came from North America, Europe, and Asia, whereas none were conducted in Africa, which may limit generalizability of the findings.

## Conclusions

Our key findings showed that the great majority of the general population has RAH, which in most cases originates from the A2 segment of ACA, most often is a single vessel and passes anteriorly in relation to ACA. Regarding our aforementioned anatomical findings, the existence of wide variations in RAH should be taken into consideration in order to avoid undesirable complications during surgeries performed in close relation to the anterior segment of the circle of Willis, therefore preventing postoperative neurological deficits.

## Supplementary information

Below is the link to the electronic supplementary material.

Supplementary file1 (17.3 KB)

## Data Availability

The authors declare that the data supporting the findings of this study are available within the paper and its supplementary information files. Data sets generated during the current study are available from the corresponding author on reasonable request.
