# Supplementary material for: The prevalence and anatomy of recurrent artery of Heubner: a meta analysis with neurosurgical considerations
Source: Acta Neurochir (Wien). 2024 Oct 29;166(1):431. doi: 10.1007/s00701-024-06327-0 (PMC11522058; doi:10.1007/s00701-024-06327-0)
Supplement: Supplementary file 1 — Supplementary file1 (17.3 KB) [file 701_2024_6327_MOESM1_ESM.docx]

**The prevalence and anatomy of recurrent artery of Heubner: a meta analysis with surgical considerations**

*Acta Neurochirurgica*

Aleksander Osiowski^1,4^, Kacper Stolarz^1,4^, Maksymilian Osiowski^1,4^, Tomasz Klepinowski^2^, Dominik Taterra^1,3,4^

Affiliations

1) Faculty of Medicine, Jagiellonian University Medical College, sw. Anny 12, 31-008 Krakow, Poland

2) Department of Neurosurgery, Pomeranian Medical University Hospital No. 1, Unii Lubelskiej 1, 71-252 Szczecin, Poland

3) Department of Orthopedics, Jagiellonian University Medical College, Balzera 15, 34-500 Zakopane, Poland

4) Ortho and Spine Research Group, Zakopane, Poland

Corresponding author:

Dominik Taterra, MD

dominik.taterra@gmail.com

Department of Orthopedics and Rehabilitation

Jagiellonian University Medical College

Zakopane, Poland

| RISK OF BIAS | | | | |
| --- | --- | --- | --- | --- |
| Study | OBJECTIVE(S) AND STUDY CHARACTERISTICS | STUDY DESIGN | METHODOLOGY CHARACTERIZATION | DESCRIPTIVE ANATOMY |
| Agosti2022 | Low | Low | High | Low |
| Agrawal2019 | Low | Low | High | Low |
| Ahmed1967 | High | Low | High | Low |
| Avci 2003 | High | Low | High | Low |
| Aydin1994 | Low | Low | High | Low |
| Boongird2009 | Low | Low | High | Low |
| Corredor2020 | Low | Low | High | Low |
| DAvella2015 | High | Low | High | Low |
| Dimitriu2013 | High | Low | High | Low |
| Dunker1976 | Low | Low | High | Low |
| ElFalougy2013 | Low | Low | High | Low |
| GascaGonzalez2017 | High | Low | High | Low |
| Gomes1984 | Low | Low | High | Low |
| Gorczyca1987 | Low | Low | High | Low |
| Kedia2013 | High | Low | High | Low |
| Lemos1976 | Low | Low | High | Low |
| Loukas2006 | Low | Low | High | Low |
| Maga2013 | Low | Low | Low | Low |
| Marinkovic1986 | High | Low | High | Low |
| Matsuda2018 | Low | Low | High | Low |
| Musso2002 | High | Low | High | High |
| Najera2019 | Low | Low | High | Low |
| Papazova2018 | Low | Low | High | Low |
| Perlmutter1976 | High | Low | High | Low |
| Rosner1984 | High | Low | High | Low |
| Tao2006 | High | Low | High | Low |
| Tulleken1978 | High | Low | High | High |
| Ugur2006 | Low | Low | High | Low |
| Uzun2009 | Low | Low | High | Low |
| Valli2021 | Low | Low | High | Low |
| Vasovic2009 | Low | Low | High | Low |
| VasquezLoayza2016 | Low | Low | High | Low |
| Zhu2021 | Low | Low | High | Low |
| Zunonkipre2011 | Low | Low | High | Low |
